# Letrozole sensitizes breast cancer cells to ionizing radiation

**DOI:** 10.1186/bcr969

**Published:** 2004-12-07

**Authors:** David Azria, Christel Larbouret, Severine Cunat, Mahmut Ozsahin, Sophie Gourgou, Pierre Martineau, Dean B Evans, Gilles Romieu, Pascal Pujol, Andre Pèlegrin

**Affiliations:** 1 Department of Radiation Oncology, CRLC Val d'Aurelle, Montpellier, France; 2 INSERM EMI0227, CRLC Val d'Aurelle, Montpellier, France; 3 INSERM U 540, Montpellier, France; 4 Department of Radiation Oncology, Lausanne, Switzerland; 5 Biostatistics Unit, CRLC Val d'Aurelle, Montpellier, France; 6 Centre for Pharmacology and Health Biotechnology, CNRS, Montpellier, France; 7 Novartis Pharma AG, Basel, Switzerland; 8 Department of Medical Oncology, CRLC Val d'Aurelle, Montpellier, France

**Keywords:** breast cancer, concurrent treatment, letrozole, radiotherapy

## Abstract

**Introduction:**

Radiotherapy (RT) is considered a standard treatment option after surgery for breast cancer. Letrozole, an aromatase inhibitor, is being evaluated in the adjuvant setting. We determined the effects of the combination of RT and letrozole in the aromatase-expressing breast tumour cell line MCF-7CA, stably transfected with the CYP19 gene.

**Methods:**

Irradiations were performed using a cobalt-60 source with doses ranging from 0 to 4 Gy. Cells were incubated with androstenedione in the presence or absence of letrozole. Effects of treatment were evaluated using clonogenic assays, tetrazolium salt colorimetric (MTT) assays, and cell number determinations. Cell-cycle analyses were conducted using flow cytometry.

**Results:**

The survival fraction at 2 Gy was 0.66 for RT alone and was 0.44 for RT plus letrozole (*P *= 0.02). Growth of MCF-7CA cells as measured by the cell number 6 days after radiotherapy (2 and 4 Gy) was decreased by 76% in those cells treated additionally with letrozole (0.7 μM) compared with those receiving radiotherapy alone (*P *= 0.009). Growth inhibition, assessed either by cell number (*P *= 0.009) or by the MTT assay (*P *= 0.02), was increased after 12 days of the combination treatment. Compared with radiation alone, the combination of radiation and letrozole produced a significant decrease in radiation-induced G_2 _phase arrest and a decrease of cells in the S phase, with cell redistribution in the G_1 _phase.

**Conclusions:**

These radiobiological results may form the basis for concurrent use of letrozole and radiation as postsurgical adjuvant therapy for breast cancer.

## Introduction

The aromatase inhibitor letrozole has been shown to be superior to tamoxifen (TAM) in the first-line treatment of metastatic breast cancer [1,2]. A randomized clinical trial comparing these two drugs in the adjuvant setting is currently ongoing, and should soon provide insight into the relative efficacy of letrozole and TAM. Another adjuvant trial, the MA.17 study, evaluated patients from the National Cancer Institute of Canada Clinical Trials Group who were disease-free after initially being treated with 5 years of TAM and then being randomized to receive either 5 years of placebo or 5 years of letrozole treatment. As compared with placebo, letrozole therapy after the completion of TAM treatment significantly improved disease-free survival [3].

Postoperative radiotherapy (RT) decreases the risk of locoregional recurrence. RT is also associated with improved survival in high-risk premenopausal and postmenopausal women with breast cancer given either adjuvant chemotherapy [4] or TAM [5], respectively. Whether letrozole sensitizes breast cancer cells to RT has not been determined. The present study has examined this question *in vitro*, using a human breast cancer cell line stably transfected with the human aromatase gene. We present herein the capacity of letrozole to sensitize these cells to RT using clonogenic, MTT, and cell-count assays. Cell-cycle analyses showed a G_1 _phase arrest and a decrease of the S phase with letrozole as compared with control cells. Moreover, compared with RT alone, combined RT and letrozole produced a significant decrease in radiation-induced G_2 _phase arrest and in the number of cells in the S phase, with cell redistribution in the G_1 _phase.

## Materials and methods

### Cell line, culture, and letrozole

MCF-7 human breast cancer cells stably transfected with the human aromatase/CYP19 gene (MCF-7CA) were kindly provided by Dr S Chen (City of Hope, Duarte, CA, USA [6]). These cells were maintained in DMEM/NUT MIX F-12 containing 10% heat-inactivated FCS (Gibco Laboratories, Cergy Pontoise, France), 500 μg/ml geneticin (G418), 300 μg/ml glutamine, 0.25 μg/ml fungizone, 100 μg/ml streptomycin, and 100 units/ml penicillin G. The two cell lines, MCF-7 wild type and MCF-7CA, were adherent and grew as monolayers at 37°C in a humidified 5% CO_2 _incubator. The cells were harvested with 0.5 g/l trypsin (Gibco Laboratories) and 0.2 g/l EDTA (Gibco Laboratories) for 3 min. Cultures were checked for the absence of mycoplasma every month.

Steroids were removed from the FCS by two treatments with dextran-coated charcoal (DCC) [7]. Cells were cultured as monolayers in 60-mm Petri dishes with phenol-red-free medium and DCC FCS.

Letrozole used in the study was synthesized in the laboratories of Novartis Pharma AG (Basel, Switzerland). In all experiments, letrozole was added to cultures that were nonconfluent at concentrations of 7 nM–0.7 μM in 10 μl ethanol 6 days before RT.

### Aromatase tritiated water assay

Aromatase activity was determined using the ^3^H_2_O release method reported by Zhou and colleagues [6], based on the procedure described by Thompson and Siiteri [8]. Cells were maintained for 5 days in 10% FCS phenol-red-free medium and then seeded at 700,000 cells per well in six-well dishes with fresh DCC-treated medium. Three days later, culture plates were washed twice with PBS. One millitre of serum-free medium containing 40 nM 1β-^3^H(N)-androst-4-ene-3,17-dione (NET-926; NEN PerkinElmer Life Sciences, Inc., Courtaboeuf, France) as substrate (specific activity, 25.9 Ci/mmol) with or without 100 nM letrozole was then added to each well. After 6 hours of incubation at 37°C, the reaction mixture was removed and extracted with two volumes of chloroform to terminate the reaction and to extract unused substrate and steroids. After 5 min centrifugation at 100 × *g*, the aqueous phase was then treated with an equal volume of 5% charcoal suspension to eliminate residual steroids. After 5 min centrifugation at 15,000 × *g*, radioactivity was assessed by liquid scintillation counting. The protein concentration was determined by the Bradford method [9] after cell extraction with Reporter Lysis 5X Buffer according to the manufacturer's instructions (PROMEGA Corp., Lyon, France). Aromatase activity was then calculated from the disintegrations per minute as picomoles of oestrogen produced by milligrams of protein per hour

### Radiation modalities

Cells were plated in 10 ml DCC and phenol-red-free medium (to ensure homogeneous energy deposition within each dish using 60-mm Petri dishes) and irradiated with a cobalt-60 source (γ irradiation, ELITE 100; Theratronics, Ottawa, ON, Canada) in the Radiation Department. The radiation was delivered as a single dose ranging from 0 to 4 Gy in an 11 cm × 11 cm field size at a dose rate of 0.5 Gy/min. A 3-cm polystyrene block was used under the Petri dishes during each irradiation to allow homogeneous backscattering γ-rays. The source-half-depth distance was initially calculated to obtain a constant dose rate of 0.5 Gy/min, and was adapted monthly to the cobalt-60 source radioactivity decay. Control cells were removed from the incubator and were placed for the same period of time under the cobalt-60 source but without RT. In the combined treatment modality studies, letrozole was added 6 days prior to RT.

### Cytotoxicity assays

Cultures were trypsinized and washed, and cells were plated in quintuplicate at a density of 100 cells per 60-mm Petri dish after dilution. Letrozole was added at concentrations ranging from 7 nM to 0.7 μM 24 hours after the cells were plated to allow for cell attachment. Cells were incubated at 37°C in a humidified chamber containing 5% CO_2 _for 12 days. The colonies were then fixed with a 1:3 (v/v) acetic acid methanol solution and were stained with 10% Giemsa (Sigma Chemical Co., St Louis, MO, USA); colonies of greater than 50 cells were scored. The plating efficiency was calculated with and without letrozole.

The cytotoxicity of RT against asynchronous, exponentially growing cells was also determined from colony formation assays. Before irradiation, the cell density was determined using appropriate dilutions (100, 100, 200, 300, and 400 cells for 0, 1, 2, 3, and 4 Gy, respectively), and cells were plated onto five replicate 60-mm Petri dishes. Cells were irradiated as already described 24 hours after plating to allow for cell attachment before the administration of RT. The letrozole-containing medium was given at a concentration of 0.7 μM 6 days before RT. Cultures were irradiated with the drug present in the medium, and were immediately returned to the incubator after irradiation. Colonies were counted after 12 days.

Experimentally derived data points are the mean of five experiments. The dose–response curves were fitted to a four-parameter model, where the response *R *varies with the dose *D *according to the equation: *R *= *a*/[1 + (*D*/*b*)^*c*^] + *R*∞, where *a *is the difference between the maximum and minimum response, *b *is the concentration of drug needed to obtain 50% of the maximal effect, *c *is a slope factor, and *R*∞ is the maximal effect. The multitarget model survival curves were fitted to the data using a least-squares regression to the linear-quadratic model: *S *= *S*_0 _exp(-α*D*_1 _- β*D*_1_^2^), where *D*_1 _is the radiation dose, *S *is the surviving fraction, and *S*_0 _is a normalizing parameter.

### MTT and cell number assays

The antiproliferative effect of letrozole with or without RT on the MCF-7CA cell line was evaluated using the MTT assay as described by Mosmann [10]. Briefly, exponentially growing cells were seeded into 96-well plates and were incubated in medium containing letrozole from 7 nM to 0.7 μM for 48 hours at 37°C. Duplicate plates containing six replicate wells/assay condition were seeded in 0.1 ml medium at densities of 5000, 15,000, and 35,000 cells per well, and were then treated with letrozole alone, with RT (2 Gy) ± letrozole (0.7 μM), and with RT (4 Gy) ± letrozole (0.7 μM), respectively. Twelve days after letrozole ± RT (2 or 4 Gy) treatment, the viability of the cells was analysed using the tetrazolium salt (Sigma) MTT colorimetric assay. Briefly, 50 μl of a 0.5% MTT solution was added to each well, followed by incubation for 4 hours at 37°C to allow MTT metabolization. The crystals formed were dissolved by adding 100 μl/well isopropylic alcohol, 1 N HCl. The absorbance at 540 nm was measured on a Microtiter^® ^Plate Reader MRX (Dynatech Laboratories, Chantilly, VA, USA). The results were expressed with respect to control values (cells without any treatment).

The antiproliferative effect of letrozole ± RT on the MCF-7CA cell line was also evaluated using a cell-count assay. MCF-7CA cells were allowed to grow for 24 hours, at which time letrozole was added at a concentration of 0.7 μM. RT (2 and 4 Gy) was delivered to those cells receiving the combination treatment 6 days after the addition of letrozole to the medium. Cells were counted every 6 days (days 6, 12, and 18) over an 18-day period after removing the cells from the plates with 0.5 g/l trypsin.

### Flow cytometry

Cells were plated in 60-mm Petri dishes at a density of 2 × 10^6 ^cells/dish. Treatment consisted of letrozole (0.7 μM) alone, RT (2 and 4 Gy) alone, or letrozole (0.7 μM) plus RT (2 and 4 Gy). Cells were collected 15 days after plating, and were processed for cell-cycle analysis. Cells were harvested by trypsinization, were washed with PBS, and then 1 × 10^6 ^cells/dish of treated cells were fixed in 70% ethanol for 2 min. After removal of ethanol by centrifugation, cells were then stained with a solution containing 40 μg/ml propidium iodide (Sigma) and 0.1 mg/ml RNase A (Roche, Indianapolis, IN, USA). Stained nuclei were analysed for DNA-PI fluorescence using a Becton-Dickinson FACScan flow cytometer (Mountain View, CA, USA). Resulting DNA distributions were analysed using the CellQUEST software (Becton Dickinson) for the proportion of cells in the sub-G_0 _phase, the G_1 _phase, the S phase, and the G_2_–M phase of the cell cycle.

In a second series of experiments, cells were treated with letrozole (0.7 μM) alone and were then cultured for 21 days. Cells were stained at different time points up to 21 days and were analysed for DNA content on a FACScan as already described.

### Statistical analysis

The nonparametric Wilcoxon signed-rank test was used to compare the surviving fraction for each dose between the two groups (RT alone and RT plus letrozole). All experiments were performed three times and the results are expressed as the mean ± standard error. All statistical tests were two-sided with an alpha level of 0.05. Data were analysed using the STATA 7.0 software (Stata Corporation, College Station, TX, USA).

## Results

### Letrozole inhibits MCF-7CA aromatase activity

**Figure 1:**
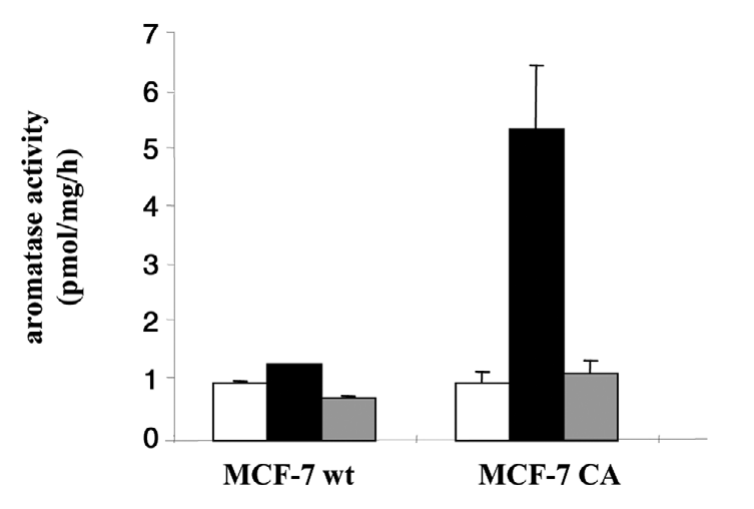
Aromatase activity in MCF-7 wild-type (wt) and MCF-7CA transfected cell lines. Aromatase activity was measured by the tritiated water assay. Treatment with letrozole inhibited the 40 nM 1β-^3^H-androstenedione substrate conversion. Open bars, filled bars, and grey bars represent background radioactivity, aromatase activity without treatment, and aromatase activity after treatment with letrozole (100 nM), respectively.

The aromatase activity was induced by the androgenic substrate Δ4 androstenedione at a 40 nM concentration. The effects of letrozole on aromatase activity in cultured breast cancer cells are shown in Fig. 1. In the MCF-7CA transfected cell line, letrozole (100 nM) markedly inhibited aromatase activity.

### Letrozole inhibits MCF-7CA proliferation

**Figure 2:**
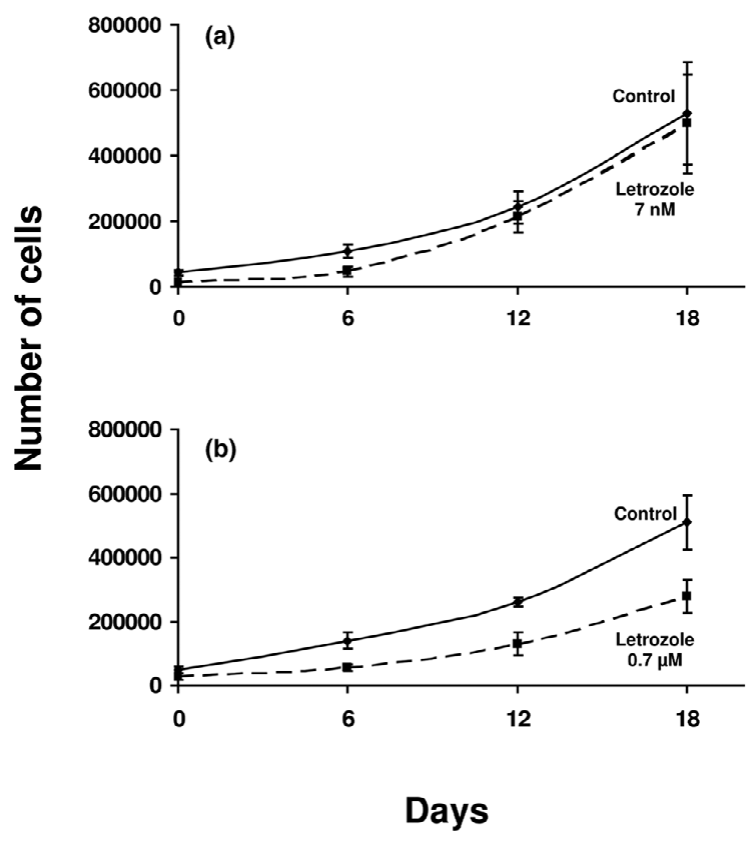
Growth inhibition with letrozole alone. **(a) **Seven nanomolar letrozole did not inhibit the growth of MCF-7CA cells during 18 days of incubation, whereas **(b) **0.7 μM letrozole resulted in about 50% inhibition after 6, 12, and 18 days of incubation. Results are from cell-count assays.

The cytotoxic effects of several concentrations of letrozole (7 nM–0.7 μM) on asynchronous, exponentially growing MCF-7CA cells were determined by cell-count assay. Letrozole at a concentration of 7 nM did not inhibit the growth of MCF-7CA cells during 18 days of incubation, whereas 0.7 μM resulted in about 50 ± 10% inhibition after 6, 12, and 18 days of incubation (Fig. 2). No effect of letrozole was seen on MCF-7 wild-type cells.

### Letrozole enhances radiosensitivity

**Figure 3:**
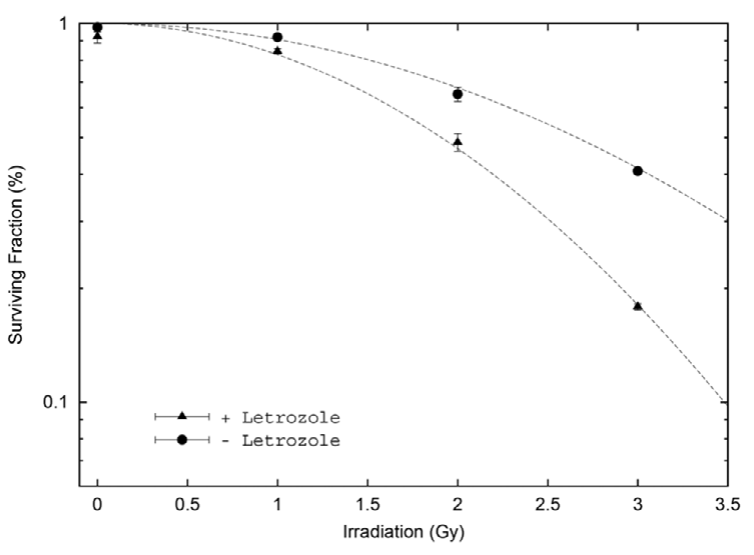
Potentiation of radiation-induced growth inhibition by letrozole measured by clonogenic assay. With radiation alone the MCF-7CA cell survival fraction decreased in a dose-dependent manner, which was significantly potentiated by the addition of 0.7 μM letrozole. For 2 Gy radiation, the surviving fraction was 0.66 with radiation alone and was 0.46 with the addition of letrozole (*P *= 0.02). For 3 Gy radiation, the corresponding surviving fractions were 0.4 and 0.18, respectively (*P *= 0.02).

Cell survival following RT in aerated medium fits a linear quadratic model, as described in Materials and methods. Figure 3 depicts normalized results from clonogenic experiments. The survival fraction at 2 Gy was 0.66 ± 0.05, and the *D*_0 _value (dose of radiation producing a 37% survival rate) was 3.2 Gy when irradiation was used alone. Letrozole added 6 days before RT led to a greater decrease of the surviving fractions as compared with those obtained when letrozole was added 3 days before RT, when letrozole was added 3 days after RT or when RT was delivered alone. Treatment at 6 days before RT led to a survival fraction at 2 Gy and a D_0 _value of 0.46 ± 0.05 and 2.2 Gy, respectively. The survival fraction at 2 Gy was thus nearly 30% (±4%) lower for the combination treatment, with a significant test result (*P *= 0.02). When the data were analysed according to the linear quadratic model, the α and β components were, respectively, nearly 0/Gy and 0.098 ± 0.0038/Gy^2 ^without letrozole, and were nearly 0/Gy and 0.154 ± 0.014/Gy^2 ^for the combination treatment. These data indicate that treatment with letrozole results in a steeper decline in cell survival due both to a higher initial slope of the dose–response curve and to a major decrease of the quadratic parameter. These results thus show possible additive effects for the combined treatment.

**Figure 4:**
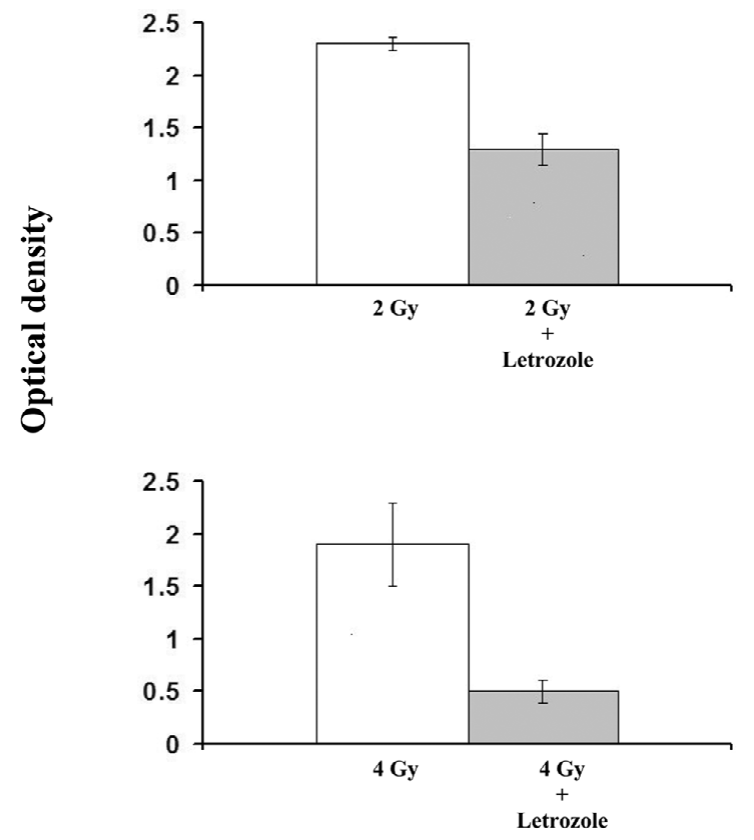
Potentiation of radiation-induced growth inhibition by letrozole measured by the MTT assay. Growth of MCF-7CA cells, measured 6 days after treatment, was inhibited to a 40% greater extent with letrozole plus 2 Gy radiation, and to a 76% greater extent with letrozole plus 4 Gy radiation, compared with radiation alone.

Growth of MCF-7CA cells measured by MTT assay was inhibited to a 40 ± 6% greater extent with letrozole plus 2 Gy RT, and to a 76 ± 3% greater extent with letrozole plus 4 Gy RT, compared with radiation alone (*P *= 0.02) (Fig. 4).

**Figure 5:**
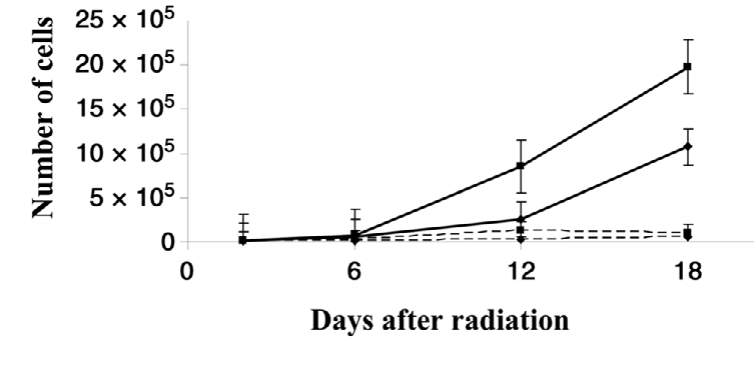
Potentiation of radiation-induced growth inhibition by letrozole measured by cell-count assay. Growth of MCF-7CA cells, measured for 18 days after treatment, was inhibited to a 76% greater extent with letrozole plus 4 Gy radiation after 12 days, and to an 85% greater extent after 18 days, compared with radiation alone. Solid lines, ■ and ◆ represent radiation alone at 2 Gy and 4 Gy, respectively; dotted lines, ■ and ◆ represent combination of radiation plus letrozole (0.7 μM) at 2 Gy and 4 Gy, respectively.

Growth of MCF-7CA cells measured by cell-count assay was determined for an 18-day period after initial treatment. It was inhibited to a 76 ± 2% greater extent after 12 days, and to an 85 ± 4% greater extent after 18 days, for letrozole treatment plus 2 or 4 Gy RT compared with RT alone (*P *= 0.009) (Fig. 5).

### Letrozole induces G_1 _cell-cycle arrest

**Figure 6:**
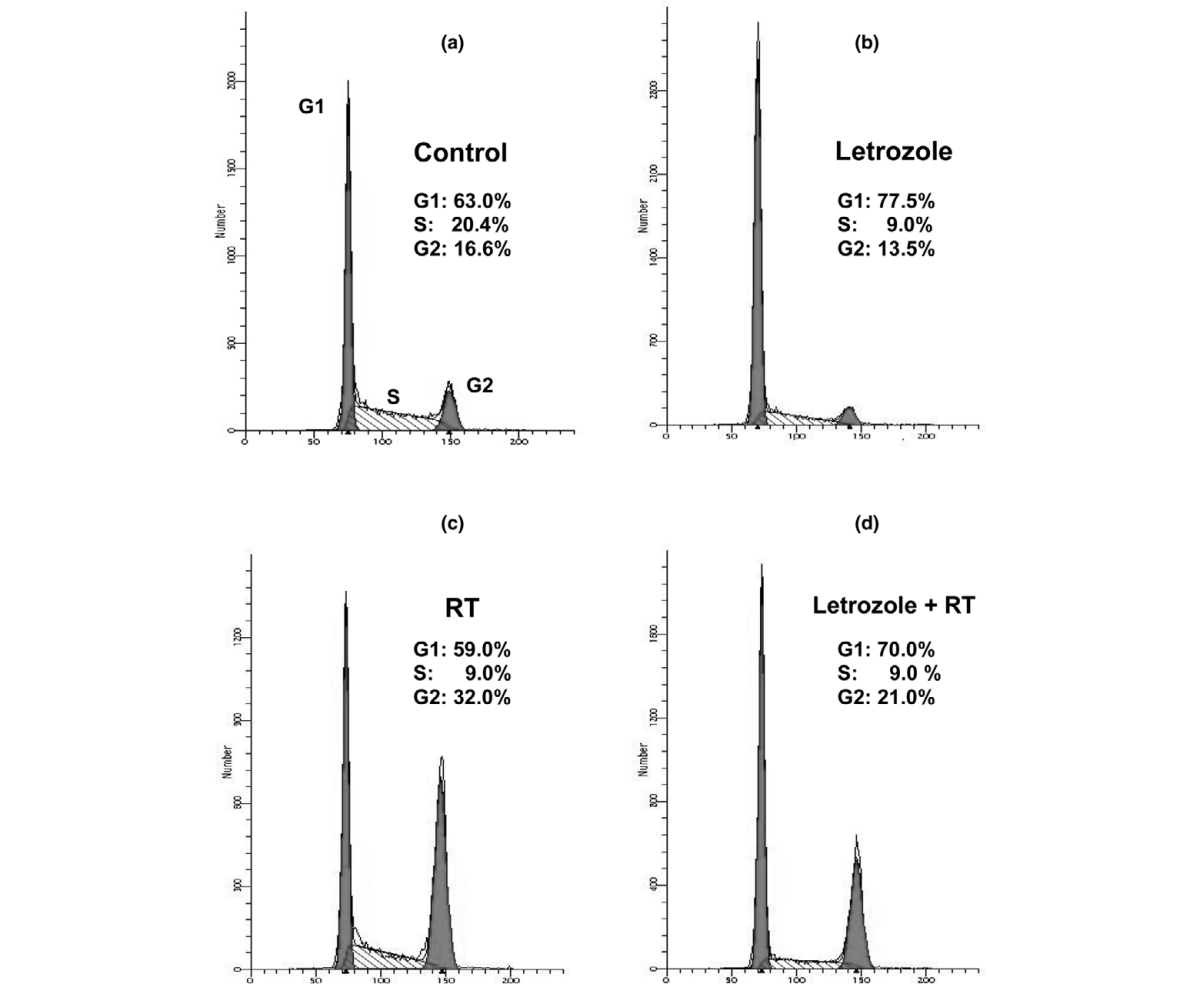
Effect of letrozole (0.7 μM) or/and radiotherapy (RT) on MCF-7CA cell-cycle progression. **(a) **Control cells were compared with **(b) **MCF-7CA cells harvested after exposure to letrozole. In the case of RT treatment, cells were harvested after RT **(c) **without or **(d) **with letrozole. Cells were fixed and stained with propidium iodide for flow cytometry analysis as described in Materials and methods. Percentages of the G_0_/G_1 _phase, the S phase, and the G_2_/M phase were determined by CellQUEST analysis software on the basis of DNA content of the histogram. Data represent mean values of duplicate samples. Similar results were obtained in replicate experiments.

The effect of letrozole treatment on cell-cycle phase distribution in the MCF-7CA cell line was evaluated using flow cytometry (Fig. 6). Treatment with 0.7 μM letrozole for 6 days induced accumulation of cells in the G_1 _phase (77.5 ± 1.5%), with a significant decrease in the percentage of cells in the S phase (9.0 ± 1%) relative to controls (20.4 ± 0.7%). No cells with subdiploid DNA content were observed, demonstrating that letrozole does not induce apoptosis in these cell lines.

After 6 days of treatment, cells were further cultured for 21 days in the presence of letrozole. A G_1 _cell-cycle arrest (nearly 70%) was observed at days 12 and 21. One day after RT alone, we observed a cell-cycle arrest in the G_2 _phase (32 ± 0.3%) with a decrease in the percentage of cells in the G_0_/G_1 _phase and the S phase as compared with the control (59 ± 0.8% versus 63 ± 1.2%, and 9 ± 0.4% versus 20.4 ± 0.7%, respectively). When letrozole was added 6 days before RT, the radiation-induced G_2_/M phase arrest decreased compared with RT alone (21 ± 0.6% versus 32 ± 0.3%, respectively), with a letrozole-induced G_0_/G_1 _phase blockade and a very low proportion of cells in the S phase.

## Discussion

TAM is the most widely used endocrine agent for the adjuvant therapy of early breast cancer in postmenopausal women [11]. TAM use nevertheless remains limited, most notably by its recognized pharmacological properties and side effects. For instance, TAM has been associated with an increased risk of both thromboembolic events and endometrial changes, including endometrial cancer [12,13]. It is possible, therefore, that other endocrine drugs may provide at least equivalent, if not superior, efficacy together with improved tolerability in patients with early disease. In this context, the third-generation aromatase inhibitors are now under investigation, and the initial results may substantially change our current clinical practice patterns [3,14,15].

The efficacy of breast-conserving surgery with axillary dissection and breast radiation has been known for a long time [16]. Overgaard and colleagues more recently demonstrated that postoperative radiotherapy decreases the risk of locoregional recurrence, and that it is associated with improved survival in high-risk patients with breast cancer given either adjuvant chemotherapy (in premenopausal women) [4] or tamoxifen (in postmenopausal women) [5].

In the present study, we wanted to evaluate *in vitro *the effects of the combination of ionizing radiation and letrozole, one of the third-generation aromatase inhibitors. To our knowledge, our results demonstrate for the first time that letrozole could act as a potent radiosensitizer. A significant effect was observed in all three assays used in our experiments (clonogenic, MTT, and cell count). The effects of associating other hormonotherapies, such as TAM, with radiotherapy have been rarely described and remain poorly defined. TAM appears to exert its cytostatic activity at least partly through competitive inhibition of oestrogen binding at the oestrogen receptor, resulting in segregation of cells into the G_0_/G_1 _phase of the cell cycle [17]. Because relatively less radiosensitivity was observed in the early G_1 _phase [18], a hypothetical concern was raised in several studies that the use of TAM during RT might result in the radioprotection of tumour clonogens of both hormonally responsive and unresponsive breast carcinoma cells at dose levels typically used in clinical practice [19]. In these studies, however, cell cultures were grown in a medium containing phenol red and foetal bovine serum, two sources of exogenous estrogenic compounds [20-23]. This fact complicates the interpretation of the resultant radiation survival curves [24]. In contrast to these reports, no significant differences were observed in radiosensitivity for oestradiol-stimulated or 4-hydroxytamoxifen-inhibited cultures plated into growth-stimulating conditions immediately after irradiation or following an additional 24 hours in oestrogen-free conditions [25]. Clearly, under defined hormonal conditions [26], no protective effect of the active TAM metabolite 4-hydroxytamoxifen was observed [25].

Our data suggest that letrozole inhibits cell proliferation by invoking a transition delay or by blocking in the G_1 _phase of the cell cycle. This delay may require more than one complete passage of some cells through the cell cycle, since cell numbers more than doubled before the plateau growth phase was reached. In addition, two to three generation times were required for maximal accumulation of cells in the G_1 _phase. We therefore added letrozole 6 days before RT. Under defined hormonal conditions, we found more than an additive effect [27] using the combination of letrozole and RT at each dose tested. This result was reinforced by the poor effect of letrozole used alone at high concentration (7 μM) and tested in all combination treatments. Isobologram analyses are planned to confirm the existence of either an additive effect or a supra-additive effect for the association of these two treatment modalities in a future study, as proposed by Steel [28].

A small percentage of cells was insensitive to letrozole, and remained in the proliferative fraction (9% in the S phase). The mechanism of unresponsiveness of these cells to letrozole will require further study. As shown with anti-oestrogens [17], these data are consistent with the hypothesis that aromatase inhibitors such as letrozole induce regression of breast cancer *in vivo *by simply blocking progression of cells through the cell cycle rather than by a direct cytotoxic effect. With cell replication inhibited, tumours might then regress because of ongoing cell shedding or death, or because of interaction with normal host defences.

In the RT–letrozole combination treatment, we observed a 50% decrease of MCF-7CA cells arrested in the G_2 _phase as compared with RT alone, a proportional redistribution in the G_1 _phase, and an interrupted synthesis phase for an 18-day period. This cell-cycle redistribution phenomenon may also explain the decrease in the surviving fraction in the combination treatment presented in the present study (Fig. 3).

In addition, associating irradiation and letrozole may modify the cytosolic oestrogen and progesterone receptor content in breast cancer cells, which might explain the observed changes in these cells' radiation sensitivity during hormonal treatment, as published with TAM [29].

These results may have important clinical implications for the treatment of breast cancer. Since the adjuvant use of aromatase inhibitors is still an emerging treatment option [3,14,15], the most effective time at which to commence the use of these drugs after surgery is not yet known. Also, if letrozole is simply placing hormone-responsive breast cancer cells into a 'resting' phase of the cell cycle (G_0_–G_1 _phase), then treatment of patients after surgery for primary breast cancer with adjuvant letrozole may have to be continued at least long enough for host defences or normal cell attrition to eradicate residual tumour cells. Premature discontinuation of therapy might lead to tumour regrowth. Finally, where letrozole is used concurrently with RT, we recommend starting letrozole at least 3 weeks before the start of RT in an attempt to obtain an optimal effect, consistent with the biological rationale described earlier. Furthermore, and in so far as the preclinical data with breast cancer cells can be extrapolated to the clinical situation, a radiosensitizing effect may be observed in the healthy breast. Nevertheless, Miller and colleagues showed a rising aromatase activity level in adipose tissue obtained from patients with breast cancer and within the quadrant involved with the tumour [30-32].

## Conclusions

These results are the first preclinical findings demonstrating the radiosensitization effect of letrozole, and thus may provide a basis for the use of combined letrozole and radiation for the adjuvant therapy in breast cancer patients.

## Abbreviations

DCC = dextran-coated charcoal; DMEM = Dulbecco's modified Eagle's medium; FCS = foetal calf serum; MTT = 3-(4,5-dimethylthiazol-2-yl)-2,5-diphenyltetrazolium bromide; PBS = phosphate-buffered saline; RT = radiotherapy; TAM = tamoxifen.

## Competing interests

The author(s) declare that they have no competing interests.

## Authors' contributions

DA initiated the project and developed all first experiments. CL confirmed all previous results and performed all FACS analyses. SC and PP performed the aromatase activity assay. MO and SG made the statistical analyses. PM constructed survival curves of the clonogenic assay. DA, DBE, GR, and AP managed scientific experiments and contributed to the elaboration of the discussion.
